# Lack of cyclical fluctuations of endometrial GLUT4 expression in women with polycystic ovary syndrome: Evidence for direct regulation of GLUT4 by steroid hormones

**DOI:** 10.1016/j.bbacli.2015.08.004

**Published:** 2015-08-28

**Authors:** Peng Cui, Xin Li, Xiaoqin Wang, Yi Feng, Jin-Fang Lin, Håkan Billig, Ruijin Shao

**Affiliations:** a Department of Integrative Medicine and Neurobiology, State Key Lab of Medical Neurobiology, Shanghai Medical College and Institute of Acupuncture Research (WHO Collaborating Center for Traditional Medicine), Institute of Brain Science, Fudan University, 200032 Shanghai, China; b Department of Physiology/Endocrinology, Institute of Neuroscience and Physiology, The Sahlgrenska Academy, University of Gothenburg, 40530 Gothenburg, Sweden; c Department of Gynecology, Obstetrics and Gynecology Hospital of Fudan University, 200011, Shanghai China; d Shanghai Key Laboratory of Female Reproductive Endocrine Related Diseases, 200011 Shanghai, China; e BIOMATCELL VINN Excellence Center of Biomaterials and Cell Therapy, Department of Biomaterials, Institute of Clinical Sciences, The Sahlgrenska Academy at University of Gothenburg, Gothenburg 40530, Sweden

**Keywords:** Glucose transport 4, Steroid hormones, Endometrium, Menstrual cycle, PCOS

## Abstract

Background

Determination of the role of steroid hormones in expression and regulation of endometrial glucose transport 4 (GLUT4) in humans is important for understanding endometrial disorders such as polycystic ovary syndrome (PCOS), a common hormone-imbalance disease.

Methods

Endometrial biopsy samples were collected from non-PCOS patients with regular menstrual cycles or with hyperplasia and from PCOS patients with or without hyperplasia. In addition, endometrial tissues from postmenopausal women were incubated with human chorionic gonadotropin (hCG, 10 IU/ml), 17β-estradiol (E2, 10 nM), progesterone (P4, 100 nM), or a combination of E2 and P4 for 24 h. The expression of GLUT4 was measured at the mRNA level using quantitative real-time polymerase chain reaction (qRT-PCR) and at the protein level using Western blot analysis and immunohistochemistry.

Results

A cyclical change in GLUT4 expression pattern was observed in non-PCOS patients, and a high level of GLUT4 expression was seen in the proliferative phase compared to the secretory phase. Low levels of GLUT4 expression were found in PCOS patients compared to menstrual cycle phase-matched non-PCOS patients, and there was no significant change in GLUT4 expression in PCOS patients during the menstrual cycle. GLUT4 was localized in both epithelial and stromal cells, with notable changes in epithelial cells. We postulate that decreased GLUT4 expression might be regulated by steroid hormones. In support of this, we showed that in cultured endometrial tissues hCG and E2 alone had no effect on GLUT4 expression. However, P4 alone and P4 in combination with E2 decreased GLUT4 expression. Compared with non-PCOS controls, PCOS patients with endometrial hyperplasia exhibited decreased GLUT4 expression in particular in the epithelial cells.

Conclusion

We conclude that P4 can induce changes in endometrial GLUT4 expression during the menstrual cycle and that abnormal hormonal conditions such as PCOS disrupt normal patterns of GLUT4 expression in endometrial cells.

## 1 Introduction

The human endometrium includes epithelial cells, stromal cells, immune cells, and blood vessels [1], and both epithelial and stromal cells are exquisitely sensitive to steroid hormone stimulation in women during the menstrual cycle [2]. In addition to estrogen and progesterone, human uterine fluid contains blood-derived glucose that is required for ATP synthesis [3], [4], and the link between glucose metabolism, implantation, embryonic development, and pregnancy has been recognized [5], [6]. There is an increasing body of evidence indicating that glucose transporters (GLUTs) are responsible for the transport of glucose across the cell membrane and that they regulate glucose utilization in tissues and cells [5]. A number of GLUTs with different tissue expression, localization, and regulation profiles have been identified in humans and rodents [5]. Among them, GLUT4 (SLC2A4) is a dynamic modulator of normal glucose homeostasis in adipose and muscle tissues, and dysfuntion of GLUT4 leads to insulin resistance and type 2 diabetes [7]. Although GLUT4 mRNA and protein has been described in human and rodent endometria and uterine stromal cells [8], [9], [10], [11], [12], the finding of detectable levels of GLUT4 mRNA and protein in uterine cells in humans and rodents under physiological and pathological conditions is somewhat puzzling [8], [13], [14], [15].

Polycystic ovary syndrome (PCOS) is the most common hormone-imbalance disorder [16], and it affects 4%–18% of adolescent and reproductive-aged women worldwide [17]. It is frequently associated with adverse impacts on female reproduction [16], [18], such as recurrent pregnancy loss [19] and irregular cycle-induced anovulation infertility [20]. It is generally believed that the failure of reproductive success in PCOS patients is in part due to endometrial dysfunction [21]. Previous studies from our group and other laboratories have shown aberrant expression of endometrial GLUT4 mRNA and protein in women with PCOS [10], [14], [22], [23], [24], [25]. While in vitro experiments have shown that testosterone decreases GLUT4 expression in human endometrial epithelial cells [26], there is no direct evidence to clarify whether 17β-estradiol (E2) or progesterone (P4) or both are involved in the regulation of endometrial GLUT4 expression under normal conditions or under disease conditions such as endometrial hyperplasia.

Given the evidence for menstrual cycle-dependent regulation of endometrial GLUT4 expression [11], we set out to elucidate the cellular pattern of endometrial GLUT4 expression in women with and without PCOS during the menstrual cycle and under conditions of endometrial hyperplasia. Furthermore, we evaluated the effects of physiological doses of E2 and P4 on endometrial GLUT4 expression in order to determine which steroid hormone might contribute to the regulation of GLUT4 expression.

## 2 Materials and methods

### 2.1 Reagents and antibodies

Human chorionic gonadotropin (hCG) was from NV Organon (Oss, Holland). 17β-estradiol (E2), progesterone (P4), and 3,3-Diaminobenzidine tetrahydrochloride (DAB) were from Sigma-Aldrich (St. Louis, MO). The Avidin-biotinylated-peroxidase complex detection system (ABC kit) was from Vector Laboratories Inc. (Burlingame, CA). All antibodies were from the following sources: GLUT4 (ab33780) antibody was from Abcam (Cambridge, UK), cytokeratin 8/18 (#4546) antibody was from Cell Signaling Technology (Danvers, MA); estrogen receptor alpha (ERα, #6F11) and progesterone receptor A/B (PRA and PRB, #16) antibodies were from Novocastra Laboratories (Newcastle, UK); and estrogen receptor beta (ERβ, PPG5/10) antibody was from AbD Serotec (Oxford, UK). β-actin (P-0130), anti-mouse IgG horseradish peroxidase (HRP)-conjugated goat (A2304), and anti-rabbit IgG HRP-conjugated goat (A0545) secondary antibodies were from Sigma-Aldrich.

### 2.2 Human tissue samples

Endometrial biopsies for in vivo studies were obtained by curettage from the Obstetrics and Gynecology Hospital of Fudan University, Shanghai, with the approval of the institutional review board. Each endometrial sample was diagnosed and staged by routine pathology analysis based on standard histological criteria [27], and the patient's last reported menstrual period was recorded at the time of collection. PCOS was diagnosed based on the Rotterdam criteria provided by the American Society for Reproductive Medicine and the European Society for Human Reproduction and Embryology [28]. All fertile women (n = 19) at the proliferative (n = 13, aged 23–44 years) or secretory phases of the menstrual cycle (n = 3, aged 30–41 years) or with hyperplasia (n = 3, aged 25–43 years) taking part in the investigation had regular menstrual cycles and showed no evidence of any pathological uterine disorder. Endometrial biopsies were obtained from PCOS patients (n = 13) at the proliferative (n = 8, aged 26–36 years) and secretory phases of the menstrual cycle (n = 2, aged 28 and 40 years) or from PCOS patients with hyperplasia (n = 3, aged 26–28 years). A diagnosis of PCOS was made if at least two of the following criteria were met: 1) oligo/anovulation, 2) signs of hyperandrogenism (i.e., hirsutism and acne) and/or biochemical measurements, or 3) enhanced ovaries (at least 12 discrete follicles of 2–9 mm in diameter in one ovary or an ovarian volume > 10 cm^3^ observed by transvaginal ultrasonography). Women with other androgen-excess disorders or specific etiologies including congenital adrenal hyperplasia, Cushing's syndrome, thyroid hormone abnormalities, hyperprolactinemia, or ovarian/adrenal tumors were excluded. All PCOS patients had no history of previous first-trimester miscarriage or pregnancy. No patients had received exogenous hormonal therapy for at least three months before the procedure. All tissue samples were washed with ice-cold RNase-free phosphate-buffered saline (PBS) and either snap-frozen in liquid nitrogen and stored at − 70 °C or fixed in 4% formaldehyde and embedded in paraffin.

Endometrial tissues from postmenopausal women (n = 7, aged 63–73 years) for in vitro studies were obtained by hysterectomy. The collection and processing steps have been described [29]. Two small pieces (3 × 5 × 5 mm), containing the endometrium and the underlying myometrium, were excised from the lower uterine cavity, and the endometrium was gently isolated. The study was approved by the Research Ethics Committee at the University of Gothenburg and was conducted at the Sahlgrenska Academy, University of Gothenburg, in accordance with the Declaration of Helsinki for medical research involving human subjects. Informed consent was obtained from all patients.

### 2.3 Endometrial tissue culture and treatment

The culture condition and methods, including reagents used, are described in [30]. After washing in PBS, endometrial tissues were cut into ~ 1 × 2-mm^2^ sections with a fine scalpel under a stereomicroscope. Tissues were treated with 10 IU/ml hCG, 10 nM E2, 100 nM P4, or a combination of 10 nM E2 and 100 nM P4, and the tissues were cultured in RPMI-1640 medium (Sigma-Aldrich) with 10% fetal calf serum and 100 IU/ml penicillin/streptomycin (GIBCO-BRL, San Francisco, CA) at 37 °C in a fully humidified 5% CO_2_ atmosphere for 24 h. The selected physiological doses of hCG, E2, and P4 were previously found to ensure the effects mediated by luteinizing hormone receptors, ERs, or PRs in human endometrial tissues in vitro [30]. E2 and P4 were dissolved in 100% ethanol at a concentration of 50 mM and added to culture medium to achieve the desired final concentration. Controls were treated with 100% ethanol. At the end of the experiments, cultured tissues were snap-frozen in liquid nitrogen and stored at − 70 °C or fixed in 4% formaldehyde and embedded in paraffin.

### 2.4 RNA extraction and quantitative real-time PCR analysis

Quantitative real-time RT-PCR (qRT-PCR) was performed with an ABI PRISM 7900 sequence detection system (Applied Biosystems, Foster City, CA). The PCR parameters were set according to the manufacturer's protocols, and amplifications were performed with a SYBR®Premix Ex Taq kit (#RR420A, Takara Bio Inc., Shiga, Japan). Tissues from the endometrium of each patient were digested with RNase-free DNase I (Ambion) to remove genomic DNA. Total RNA was isolated from individual tissues using Trizol Reagent (Life Technologies) according to the manufacturer's instructions. Single-stranded cDNA was synthesized from each sample (1 μg) with PrimeScript RT Master Mix (#RR036A, Takara Bio Inc.). cDNA (1 μl) was added to a reaction master mix (10 μl) containing 2 × SYBR Green PCR reaction mix (Takara Bio Inc.) and gene-specific primers (10 μM each of forward and reverse primers). For each sample, duplicate reactions were conducted in 384-well plates. All primers (Table 1) were designed to exclude the amplification of genomic DNA. Amplification quality was validated by analysis of the melting curve. All reactions were performed in duplicate, and each reaction included a non-template control. The CT values for both ACTB (β-actin) and CYC1 (Cytochrome c isoform 1) were not significantly different in any of the groups, which confirmed that the loading was similar between the samples. The result for GLUT4 gene is expressed as the amount relative to the average value of β-actin + CYC1 in each sample. Relative gene expression was determined with the 2^−(∆∆CT)^ method.

### 2.5 Western blot analysis

Endometrial tissues were lysed using RIPA buffer (Sigma-Aldrich) supplemented with cOmplete Mini protease inhibitor cocktail tablets (Roche Diagnostics, Mannheim, Germany) and PhosSTOP phosphatase inhibitor cocktail tablets (Roche Diagnostics). After incubation for 15 min on ice, tissue lysates were cleared by centrifugation at 10,000 ×* g* for 30 min at 4 °C, and the protein concentration of the supernatant was determined with a Direct Detect® spectrometer (EMD Millipore Corporation, Billerica, MA). A detailed explanation of the Western blot analysis protocol has been published elsewhere [31]. Equal amounts of protein for each treatment group were resolved on NuPAGE 4–12% Bis–Tris gels (Invitrogen) and transferred onto PVDF membranes. The membranes were probed with the primary antibody (1:1000–2000 dilution) of interest in 0.01 M Tris-buffered saline supplemented with Triton X-100 (TBST) containing 5% nonfat dry milk followed by HRP-conjugated secondary antibody. When necessary, PVDF membranes were stripped using Restore PLUS Western blot stripping buffer (Thermo Scientific, Rockford, IL) for 15 min at room temperature, washed twice in TBST, and then reprobed.

### 2.6 Immunohistochemistry

Immunohistochemistry was based on the previously described methodology [32]. The tissues were fixed in 4% formaldehyde neutral-buffered solution for 24 h at 4 °C. After deparaffinization and rehydration, the sections were immersed in epitope retrieval buffer (10 mM sodium citrate buffer, pH 6.0) and heated in a 700 Watt microwave for 10 min. The sections were subsequently rinsed twice with dH_2_O and once with TBST. The endogenous peroxidase and nonspecific binding were removed by incubation with 3% H_2_O_2_ for 10 min and with 10% normal goat serum for 1 h at room temperature. After incubation with the GLUT4 primary antibody (1:100 dilution) overnight at 4 °C in a humidified chamber, sections were stained using the avidin-biotinylated-peroxidase ABC kit according to the manufacture's instruction (Vector Laboratories) followed by a 5-min treatment with DAB-Ni (SK-4100, Vector Laboratories). Sections were imaged on a Nikon E-1000 microscope (Japan) under bright field optics and photomicrographed using Easy Image 1 (Bergström Instrument AB, Sweden).

### 2.7 Statistical analysis

Results are presented as means ± SEM. Statistical analyses were performed using SPSS version 21.0 statistical software for Windows (SPSS Inc., Chicago, IL). For the in vivo studies, unpaired Student's *t*-test was used to compare two groups. For the in vitro studies, data were analyzed using one-way ANOVA followed by Dunnett's post-hoc tests. A p-value less than 0.05 was considered statistically significant.

## 3 Results and discussion

**Fig. 1:**
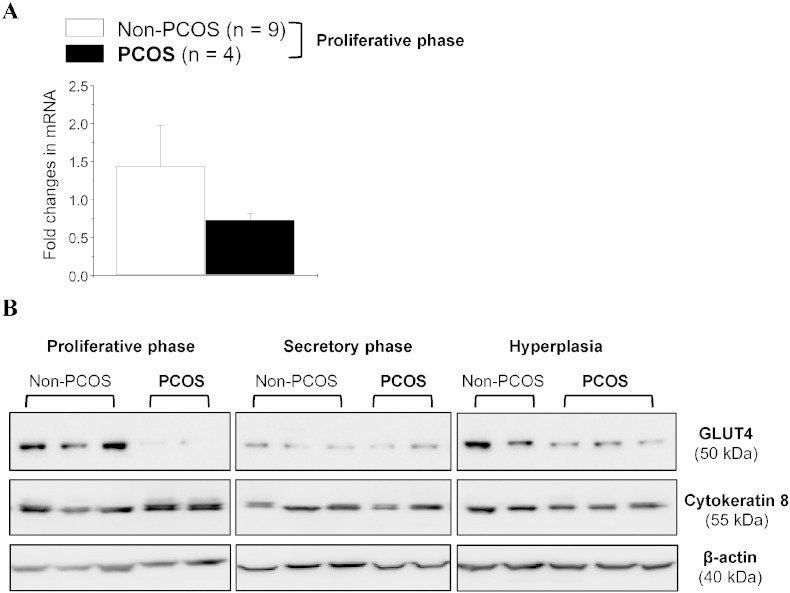
Expression of GLUT4 mRNA and proteins in the endometrium from non-PCOS and PCOS patients. Endometrial homogenates were prepared from women with and without PCOS, and qRT-PCR and Western blot assays were performed as described in the Materials and Methods. (A) Quantitative RT-PCR analysis of GLUT4 mRNA levels in the proliferative phase between non-PCOS and PCOS patients. RNA levels were normalized to the average level of ACTB (β-actin) and CYC1 (cytochrome c isoform 1). Values are the mean ± SEM. N.S., nonsignificant (p > 0.05). (B) Representative Western blot analysis of GLUT4 expression in human endometrial tissues in vivo. The level of GLUT4 protein was decreased in PCOS patients regardless of whether hyperplasia was present. Cytokeratin 8 (an epithelial cell marker) and β-actin were used as the internal controls.

**Fig. 2:**
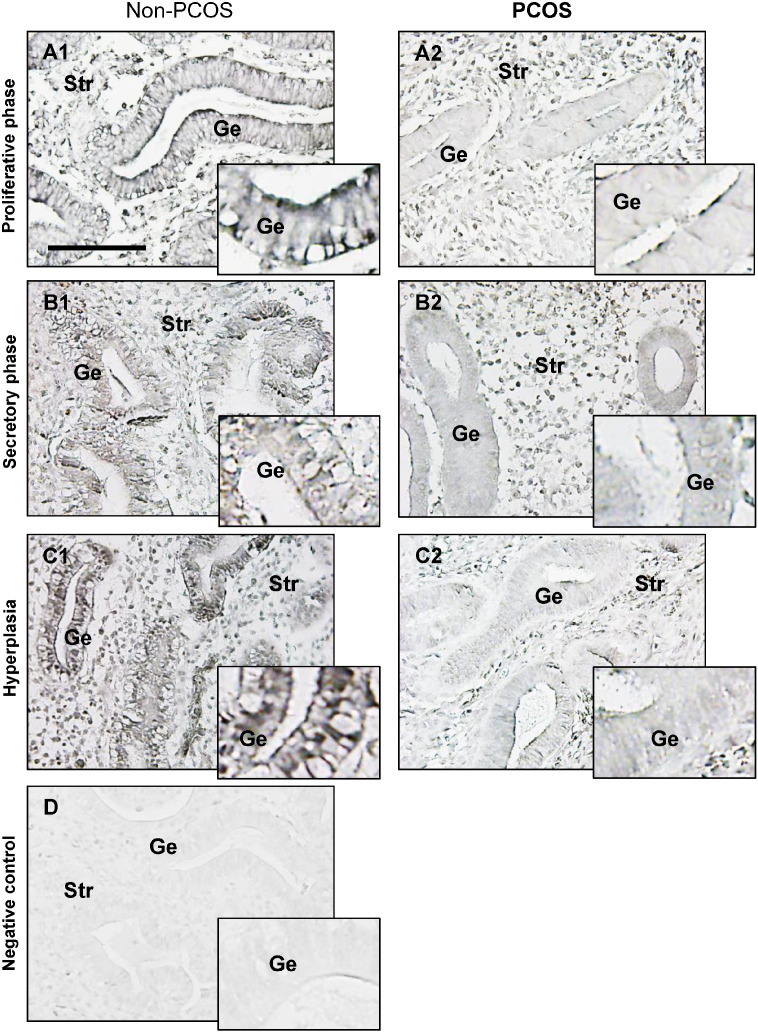
Comparison of immunohistochemical staining of GLUT4 in the endometrium from non-PCOS and PCOS patients. Representative paraffin-embedded endometrial sections in the proliferative phase of women without PCOS (A1) and with PCOS (A2), in the secretory phase of women without PCOS (B1) and with PCOS (B2), and in women with hyperplasia without PCOS (C1) and in women with both PCOS and hyperplasia (C2). The same concentration of rabbit IgG instead of the primary and secondary antibodies was used as the negative control (D). Enhanced magnifications are shown in the bottom right corner of A1–D. Immunohistochemistry was performed as described in the Materials and Methods. GLUT4 was found in all endometrial cell types, and specific immunostaining of GLUT4 (DAB-Ni-black) was detected mainly in the membrane and cytoplasm. The images are representative of those observed in numerous sections from multiple endometrial tissues. Scale bar = 100 μm. Ge, glandular epithelial cells; Str, stromal cells.

Menstrual dysfunction is a major cause of infertility [33], and menstrual cycle irregularities and disturbances are the key feature of PCOS [16], [18]. We showed that endometrial GLUT4 expression is higher in the proliferative phase than the secretory phase of the menstrual cycle in non-PCOS patients (Fig. 1B), which is in accordance with a previous report [11]. In the proliferative phase, a significant reduction in endometrial GLUT4 protein (Fig. 1B) but not mRNA (Fig. 1A) expression was observed in PCOS patients compared to non-PCOS patients. Moreover, only endometrial GLUT4 protein expression was being shown as constant throughout the menstrual cycle in PCOS patients (Fig. 1B and 2). This indicates that in non-PCOS women, different hormone environments during the menstrual cycle influence endometrial GLUT4, in contrast to women with PCOS.

**Fig. 3:**
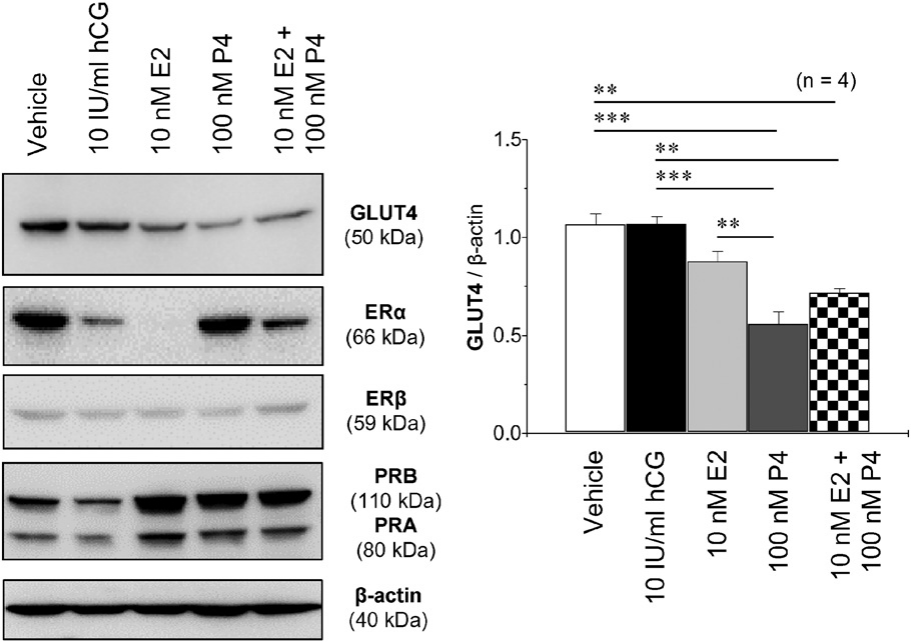
Effects of gonadotropin and steroid hormones on GLUT4 protein levels in cultured human endometrial tissues. Endometrial biopsy samples from postmenopausal women were treated with vehicle, hCG, E2, P4, or E2 + P4 for 24 h. Tissue lysates were directly immunoblotted with antibodies to GLUT4, ERα, ERβ, PRA, PRB, and β-actin as indicated. The Western blot assay was performed as described in the Materials and Methods. β-actin was used as an internal control. Values are the mean ± SEM. ** p < 0.01; *** p < 0.001. hCG, human chorionic gonadotropin; E2, 17β-estradiol; P4, progesterone; ERα, estrogen receptor alpha; ERβ, estrogen receptor beta; PRA, progesterone receptor A; PRB, progesterone receptor B.

It is well established that steroid hormones (E2 and P4) and their nuclear receptors (ERα, ERβ, PRA, and PRB) [34], [35] are tightly regulated during the menstrual cycle [2]. Furthermore, it is increasingly apparent that E2 and P4 can exert their effects through cell-membrane bound ERs and PRs. Indeed, several membrane-bound ERs [36], [37] and PRs [38], [39] have been reported to be expressed in human endometrium. The regulatory pattern for endometrial GLUT4 expression in women during the menstrual cycle has been established [11], so we next examined whether E2 and/or P4 regulates endometrial GLUT4 regulation. In the cultured endometrial tissues, Western blot analysis (Fig. 3) showed that E2 decreased ERα expression, increased PRA and PRB expression, and had no effect on GLUT4 and ERβ expression. In contrast, P4 decreased GLUT4 expression and increased ERα and PRA/B expression. Additionally, the combined treatment with E2 and P4 had a similar effect on GLUT4 expression as P4 alone. P4 has previously been shown to inhibit insulin-regulated glucose uptake by decreasing GLUT4 expression in 3T3-L1 adipocytes [40]. Previous studies have also shown that E2 and ER agonists decrease GLUT4 expression in 3T3-L1 adipocytes [41], [42] in contrast to human skeletal muscle cells [43]. Moreover, treatment with testosterone decreases GLUT4 protein expression in human endometrial epithelial cells in vitro [26]. Together, these data suggest that regulation of GLUT4 expression is steroid hormone-specific and tissue/cell-specific under physiological conditions.

Because the proliferation, differentiation, secretion, and apoptosis of endometrial cells are controlled by steroid hormones [2], and given the fact that glucose uptake and utilization play a crucial role in implantation, embryonic development, and pregnancy [5], [6], it remains to be determined how endometrial GLUT4 contributes to glucose metabolism in response to P4 stimulation under physiological conditions in vivo. On the other hand, the observation that decreased endometrial GLUT4 expression by P4 stimulation is surprising, especially because the endometria in PCOS patients tend to remain in a proliferative state due to the lack of counterbalance by P4 [44]. It has been shown that insulin/insulin receptor signaling has a metabolic function and favors glucose uptake in the endometrial cells [11], [45]. Although the molecular mechanisms underlying insulin resistance in PCOS remain elusive, insulin resistance in these patients is believed to contribute to endometrial dysfunction [16], [46]. Because GLUT4 is an insulin-responsive glucose transporter [5], and because therapeutic insulin sensitizers such as metformin increase endometrial GLUT4 expression in vivo and in vitro [10], [22], our current findings support the view that the regulation of endometrial GLUT4 expression does not depend solely on steroid hormones in PCOS patients. Understanding the specific effects of insulin on endometrial GLUT4 expression will required additional investigation.

**Fig. 4:**
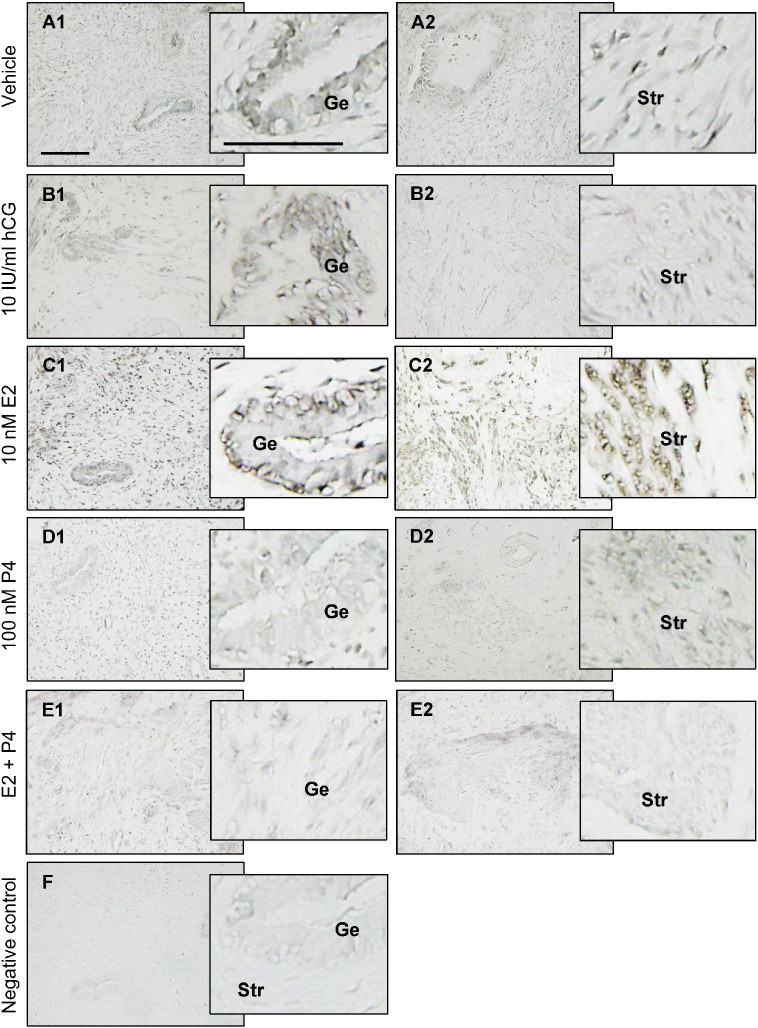
Comparison of immunohistochemical staining for GLUT4 in cultured human endometrial tissues. Endometrial biopsy samples from postmenopausal women were treated with vehicle (A1, A2), hCG (B1, B2), E2 (C1, C2), P4 (D1, D2), or E2 + P4 (E1, E2) for 24 h, fixed in formalin, and embedded in paraffin. The same concentration of rabbit IgG instead of the primary and secondary antibodies was used as the negative control (F). Enhanced magnifications are shown on the right of A1–F. Immunohistochemistry was performed as described in the Materials and Methods. Specific immunostaining of GLUT4 (Ni-black) was detected mainly in the membrane and cytoplasm. Representative images (n = 3 patients) from three independent experiments are shown. Scale bar = 100 μm. hCG, human chorionic gonadotropin; E2, 17β-estradiol; P4, progesterone; Ge, glandular epithelial cells; Str, stromal cells.

Although the expression of GLUT4 mRNA and protein in endometrial stromal cells under physiological conditions is more controversial [8], [13], [23], our immunohistochemistry and immunofluorescence analysis [10] showed that GLUT4 immunoreactivity was present in human endometrial stromal cells under physiological conditions. We also showed that in addition to epithelial cells, stromal GLUT4 immunoreactivity was detectable and variable in cultured human endometrial tissues treated with hCG, E2, and P4 (Fig. 4). Moreover, a previous in vitro study indicated that GLUT4 protein levels can be detected by Western blot analysis in human endometrial stromal cells [47]. Based on our finding that GLUT4 expression was decreased in both epithelial and stromal cells in response to P4 alone and in combination with E2, we speculate that GLUT4 plays a unique role in endometrial epithelial and stromal cells.

A prospective cross-sectional study showed that hyperinsulinemia is often associated with endometrial hyperplasia [48]. Extensive evidence in the literature indicates that both insulin resistance and consequent hyperinsulinemia have an important role in the pathogenesis of PCOS [16], [18]. Our Western blot analysis (Fig. 1B) showed that endometrial GLUT4 expression was lower in PCOS patients than non-PCOS patients with hyperplasia. In addition, immunohistochemistry (Fig. 2) indicated that PCOS rather than hyperplasia conditions appear to have a major effect on GLUT4 expression in epithelial cells. While PCOS patients with endometrial hyperplasia are often associated with progesterone and insulin resistance [16], [18], [44], our data suggest that aberrant expression of GLUT4 might be useful in distinguishing PCOS patients with endometrial hyperplasia from non-PCOS patients with endometrial hyperplasia.

Before we conclude some of the major implication of our findings, a limitation of the study needs to mention. Our study has included the relatively small sizes of the patient groups. Thus future randomized trials with a larger sample size are needed to confirm our findings.

## 4 Conclusion

PCOS is a complex and heterogeneous disease process that involves different pathophysiological mechanisms [16], [46]. Although our patient cohort was small and a larger study is needed for validation, our study provides in vivo and in vitro evidence showing that endometrial GLUT4 expression depends on the menstrual cycle phase and the presence of PCOS. Endometrial cells can be targeted for regulating GLUT4 expression under different hormonal conditions, and we hypothesize that steroid hormone-dependent regulation of GLUT4 expression might be part of the mechanism behind PCOS-induced endometrial dysfunction. Therefore, it will be important in future studies to elucidate cellular and molecular mechanism underlying specific hormone-regulated GLUT4 expression in human endometrium.

## Competing interests

The authors indicate no potential conflicts of interest.

## Transparency document

Transparency document

## Figures and Tables

**Table 1:** Primers used for quantitative real-time PCR analyses.

Gene		Primer	Size	Reference
*SLC2A4* (GLUT4)	Forward	ATCCTTGGACGATTCCTCATTGG	90 bp	This study
Reverse	CAGGTGAGTGGGAGCAATCT
*SLC2A4* (GLUT4)	Forward	GCCATGAGCTACGTCTCCATT	90 bp	This study
Reverse	GGCCACGATGAACCAAGGAA
*SLC2A4* (GLUT4)	Forward	CTACAATGAGACGTGGCTGG	160 bp	This study
Reverse	CCTTCCAAGCCACTGAGAGA
*SLC2A4* (GLUT4)	Forward	TGCAGTTTGGGTACAACATTGG	190 bp	[13]
Reverse	ATGAGGAAGGAGGAAATCATGC
*SLC2A4* (GLUT4)	Forward	GCCCGAAAGAGTCTAAAG	407 bp	[25]
Reverse	AGAGCCACGGTCATCAAG
*ACTB* (β-actin)	Forward	CATGTACGTTGCTATCCAGGC	250 bp	This study
Reverse	CTCCTTAATGTCACGCACGAT
*CYC1* (Cytochrome c isoform 1)	Forward	AGCTATCCGTGGTCTCACC	225 bp	This study
Reverse	CCGCATGAACATCTCCCCATC
